# Acute alterations in blood lactate in the setting of transient stress induced myocardial ischaemia

**DOI:** 10.1113/EP092429

**Published:** 2025-06-02

**Authors:** Jamie M. O'Driscoll, Elliot Smith, Matchel Bibat, Jamie J. Edwards, Claire Compton, Konstantina Kipourou, Damian Coleman, Jonathan Wiles, Eliane Cunliffe, Anna Marciniak, Rajan Sharma

**Affiliations:** ^1^ Department of Cardiology St George's University Hospitals NHS Foundation Trust Tooting, London UK; ^2^ Diabetes Research Centre, College of Life Sciences University of Leicester Leicester UK; ^3^ Department of Applied Sport and Exercise Science University of East London London UK; ^4^ School of Psychology and Life Sciences Canterbury Christ Church University Canterbury UK

**Keywords:** adverse event, blood lactate, myocardial ischaemia

## Abstract

An elevation in resting venous blood lactate ([La^−^]_b_) levels in conditions of myocardial hypoperfusion is associated with adverse prognosis and survival. This investigation aimed to assess changes in venous [La^−^]_b_ levels induced by dobutamine stress in the presence and absence of myocardial ischaemia and adverse outcomes at 1 year. Four hundred and four consecutive patients (mean age 70 ± 10 years, 243 male) reporting chest pain underwent dobutamine stress echocardiography (DSE) and were categorised as ischaemic (IS) or non‐ischaemic (NI) responders. Conventional and global longitudinal strain (GLS) echocardiographic measures were recorded at rest. Venous [La^−^]_b_ samples were acquired at rest, peak stress and 1, 3, 5 and 10 min into recovery using a commercially available Lactate Pro 2 device. There were no significant differences in [La^−^]_b_ concentrations between IS (1.75 ± 0.76 mmol L^−1^) and NI (1.73 ± 0.60 mmol L^−1^) responders at baseline (*P* = 0.592). However, [La^−^]_b_ concentrations were significantly greater at peak stress (1.83 ± 0.57 vs. 1.68 ± 0.60 mmol L^−1^), 1 (1.90 ± 0.56 vs. 1.73 ± 0.71 mmol L^−1^), 3 (1.97 ± 0.56 vs. 1.73 ± 0.71 mmol L^−1^), 5 (1.98 ± 0.60 vs. 1.74 ± 0.70 mmol L^−1^) and 10 min (2.01 ± 0.63 vs. 1.76 ± 0.71 mmol L^−1^) into recovery between IS and NI responders (all *P *< 0.001). GLS was significantly lower in IS compared to NI (−15.5 ± 2.9 vs. −16.2% ± 2.7%, *P* = 0.02) responders at baseline. In patients who experienced an adverse cardiac event during 1 year of follow‐up, GLS (−14.4 ± 2.7 vs. −16.1% ± 2.8%, *P *< 0.001) and [La^−^]_b_ concentrations were significantly lower at baseline (1.54 ± 0.55 vs. 1.78 ± 0.70 mmol L^−1^, *P* = 0.02), as were [La^−^]_b_ concentrations at 5 (1.68 ± 0.55 vs. 1.88 ± 0.68 mmol L^−1^, *P* = 0.04) and 10 min (1.70 ± 0.56 vs. 1.93 ± 0.71 mmol L^−1^, *P* = 0.02) into recovery compared to patients who did not experience an adverse event. GLS (hazard ration (HR) 1.21; 95% CI: 1.11–1.33, *P *< 0.001) and [La^−^]_b_ concentrations at 10 min into recovery (HR 0.54; 95% CI: 0.33–0.85, *P* = 0.01) were significant independent predictors of an adverse event. Transient myocardial ischaemia is associated with a significant elevation in [La^−^]_b_ concentrations, which extends into the recovery period, compared to NI responders. A blunted metabolic response to dobutamine stress and attenuated longitudinal myocardial mechanics are independently associated with short‐term adverse events.

## Abstract (highlights)

- **What is the central question of this study?**
  Elevation in resting venous blood lactate ([La^−^]_b_) levels in conditions of myocardial hypoperfusion is associated with adverse prognosis and survival: what changes occur in venous [La^−^]_b_ levels induced by dobutamine stress in the presence and absence of myocardial ischaemia and what adverse outcomes occur at 1 year?
- **What is the main finding and its importance?**
  Blood lactate responses were significantly elevated in those with inducible myocardial ischaemia compared to a relatively stable response in non‐ischaemic responders. Blood lactate response to dobutamine stress independently predicted an adverse outcome at 1 year of follow‐up.

## 1 INTRODUCTION

An alteration in cardiac metabolism is the primary pathophysiological consequence of myocardial ischaemia. Interrupted blood flow and subsequent inadequate oxygen availability to the myocardium as a result of coronary vessel disease leads to anaerobic metabolism with resultant blood lactate ([La^−^]_b_) production (Parker et al., 1969). The reported shift towards a more acidic pH in the coronary sinus blood, a drop in coronary sinus oxygen saturation and altered myocardial lactate metabolism have all been used as metabolic markers of myocardial ischaemia (Rosano et al., 1996). This is associated with poor outcome.

Biochemical evidence of myocardial ischaemia is difficult to quantify during physiological exercise stress, primarily due to metabolic contributions from other organs, such as skeletal muscle (Parker et al., 1969). As such, historical research has used invasive measures (atrial pacing) to study changes in myocardial metabolism, which has reported significant reductions in pH in ischaemic (IS) responders, with no change in the non‐ischaemic (NI) responders (Cobbe & Poole‐Wilson, 1982). In addition, historical research relied upon electrocardiographic evidence of myocardial ischaemia, which is known to have poor sensitivity and specificity for detecting coronary artery disease (CAD) (Megnien & Simon, 2009). Assessment of myocardial ischaemia solely by increasing heart rate and force of contraction using dobutamine infusion, makes it possible to examine left ventricular (LV) function and myocardial metabolism without the haemodynamic and metabolic changes that are associated with exercise. Dobutamine stress echocardiography (DSE) has high accuracy (sensitivity 95.4% and specificity 96%) for the detection of CAD (Woodward et al., 2022) and allows assessment of cardiac structure, function and myocardial mechanics. Combined with DSE, it is unknown if transient myocardial ischaemia is associated with significant changes in [La^−^]_b_ or whether [La^−^]_b_ measures provide additional diagnostic or prognostic utility in patients with suspected CAD. As such, this large observational study aimed to assess alterations in [La^−^]_b_ concentrations in response to dobutamine stress in the presence and absence of myocardial ischaemia and 1 year adverse event outcomes.

## 2 METHODS

### 2.1 Ethical approval

This study was reviewed and approved by the local ethics committee (London–Surry Borders Research Ethics Committee: IRAS ID:251238) and conformed to the *Declaration of Helsinki*. Before data collection, all participants were informed about the experimental procedures and gave their written informed consent.

### 2.2 Study design and patients

**FIGURE 1:**
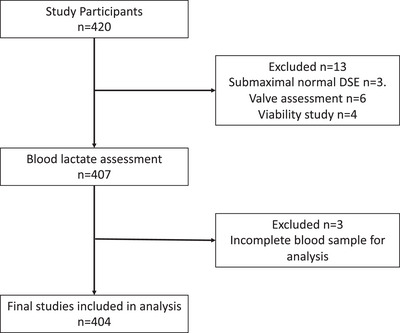
Study flow diagram.

The study dataset consisted of 404 participants fulfilling the exclusion and inclusion criteria who underwent a clinically indicated DSE for investigation of chest pain at St George's University Hospital NHS Foundation Trust (see Figure 1). Exclusion criteria included sub‐maximal haemodynamic stress (defined as not reaching the target heart rate of 85% of the age‐predicted maximal heart rate, which was calculated as 220 − age) in the absence of wall motion abnormalities (WMA), asymptomatic patients awaiting non‐cardiac surgery, and participants referred only for the assessment of myocardial viability and severity of valvular heart disease.

### 2.3 Blood lactate

**FIGURE 2:**
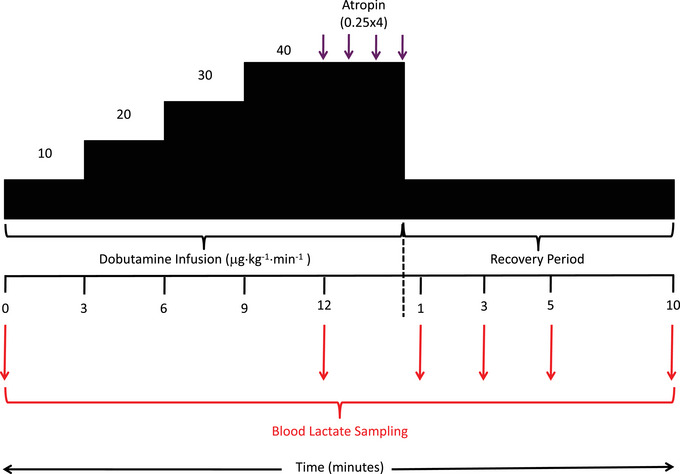
DSE protocol with timings of blood lactate sampling. DSE, dobutamine stress echocardiography.

Venous blood lactate samples were acquired using a commercially available Lactate Pro 2 device (Arkray, Kyota, Japan). Blood samples were drawn at baseline (following 15 min of supine rest), peak stress and 1, 3, 5 and 10 min after cessation of dobutamine infusion from a venous cannula. Prior to obtaining a blood sample, the cannular was flushed with 10 mL of saline. The first 5 mL blood sample drawn from the cannula was discarded, with the second 5 mL sample used for analysis. Each blood sample was presented to the testing strip of the Lactate Pro 2 at a 90 degree angle to allow the test strip to draw up blood until the check window was filled (Figure 2). Previous work has reported high reliability of the Lactate Pro 2 analyser with a coefficient of variation of 3.3% and Bland–Altman 95% limits of agreement of ±0.3 mmol L^−1^ for [La^−^]_b_ concentrations ≤4.0 mmol L^−1^ (Crotty et al., 2021).

### 2.4 Transthoracic echocardiogram

All patients underwent a full cross‐sectional transthoracic echocardiogram prior to their DSE. Image acquisitions and measurements were performed as recommended by the American Society of Echocardiography (Lang et al., 2015) and stored for offline analysis using commercial software (EchoPAC, V202, GE Healthcare). LV volumes and LV ejection fraction (LVEF) were obtained by performing endocardial tracings and using the biplane method of disks (modified Simpson's rule). Pulsed Doppler was used to record transmitral flow in the apical four‐chamber view. Tissue Doppler velocities were acquired at the septal and lateral mitral annulus, with LV filling pressure estimated from the mitral *E*/*E*′ ratio. Two‐dimensional speckle tracking imaging was utilised to calculate LV longitudinal strain from the apical two‐, three‐ and four‐chamber views. Global longitudinal strain (GLS) was calculated only when strain values were calculated from all apical views. The highest quality images were used for tracing the endocardium and a full‐thickness myocardial region of interest was selected to ensure effective application of speckle tracking analysis. All images were reviewed and excluded if any failed to meet the required optimisation and standardisation. Images were optimised for scan depth and sector width to obtain high frame rates (>60 Hz). The trace line of the endocardium and/or region‐of‐interest width was readjusted to ensure an adequate tracking score.

### 2.5 DSE

All patients recruited underwent DSE. The image quality obtained was interpretable in all patients (287 (71%) requiring contrast) and the entire cohort was used in data analysis. DSE was performed according to a standard protocol with dobutamine infusion starting at and increasing every 3 min with 10 µg kg^−1^ min^−1^ to a maximum of 40 µg kg^−1^ min^−1^ (stage 4). If no end‐point was reached, atropine was used. Mean dobutamine dose was 30.1 ± 4.9 µg kg^−1^ min^−1^ and 226 (55.9%) patients required atropine (0.53 ± 0.3 mg) to achieve target heart rate. Transthoracic echo images of the heart were acquired in standard parasternal long‐ and short‐axis and apical two‐, three‐ and four‐chamber views at baseline and during stepwise infusion of dobutamine. Baseline, low‐dose (heart rate 10–15 beats above baseline), peak and recovery (10 min post‐drug infusion) stage images were acquired as digital full cardiac cycle loops in a quad screen format and stored for off‐line analysis. The LV was divided into a 17‐segment model for qualitative analysis (Cerqueira et al., 2002) and wall motion was scored on a 4‐point scale (1, normal wall motion; 2, hypokinesis; 3, akinetic; and 4, dyskinetic) and used to calculate the wall motion score index (WMSI) at rest and peak stress (Pellikka et al., 2020). An abnormal (IS) response was defined as the worsening of wall motion under stress compared to resting function and the IS burden was categorised as low (1–2 IS LV segments) or moderate to severe (≥3 IS LV segments) (Maron et al., 2020). Non‐viable myocardium was defined as resting akinetic or dyskinetic LV segment without improvement during DSE (Rizzello et al., 2006) and referred to as fixed WMA. Interpreters of the DSE examination (A.M. and R.S.) were blinded to [La^−^]_b_ results.

### 2.6 Participant follow‐up and outcomes

For all participants, clinical follow‐up information for 12 months after the DSE was obtained from blinded review of medical (hospital and GP) records. The principal end‐point of interest for this analysis was a major adverse cardiac event (MACE) with patients censored at the time of event or at the last follow‐up. A MACE was defined as the composite of total death, non‐fatal myocardial infarction (NFMI), stroke and hospitalization because of heart failure. A NFMI was defined by the standard criteria of IS chest pain associated with an elevation of cardiac enzymes with or without electrocardiographic changes. In patients who underwent coronary angiography following DSE, significant CAD was defined according to ≥50% left main disease and/or ≥70% luminal narrowing in the left anterior descending, circumflex or right coronary artery by visual assessment and/or fractional flow reserve.

### 2.7 Data analysis

Unless otherwise specified, data are presented as means ± SD or *n* (%). Group comparisons were performed using Student's *t*‐test or the Mann–Whitney *U*‐test for continuous data and categorical data were compared with the chi‐squared (χ^2^) test. Differences between and within groups for [La^−^]_b_ concentrations were determined by two‐way repeated measures ANOVA. A Kruskal–Wallis test was used to assess differences between IS and NI responders with and without adverse events during follow‐up, at the 10 min recovery time point.

The relationship between clinical characteristics, [La^−^]_b_ concentrations, echocardiography results and clinical outcomes were evaluated using multivariable Cox regression analyses. Variable selection for the multivariable models was conducted using forward stepwise regression using a cut‐off of 0.05 for *P*‐values. Hazard ratios (HR) and corresponding 95% confidence intervals (CI) are reported.

All analyses were conducted using SPSS Statistics 26 (IBM Corp., Armonk, NY, USA). A *P*‐value < 0.05 was reported as statistically significant.

## 3 RESULTS

### 3.1 General

In total, 404 participants were included in our final analysis. The DSE examination result was interpreted as positive for inducible myocardial ischaemia in 184 (45.5%) patients and negative in 220 (54.5%). There were no significant differences between NI and IS patients with respect to age, body surface area, hypertension, diabetes, family history of cardiovascular disease, previous coronary artery bypass graft surgery or smoking history (Table 1). However, male sex, hypercholesterolaemia, previous myocardial infarction and previous percutaneous coronary intervention were more common in IS responders. In addition, IS responders were more likely to be prescribed angiotensin convertor enzyme inhibitors, aspirin, nitrates and statin therapy (Table 1). In patients who had an adverse event at 1 year of follow‐up, there were no significant differences in demographic or cardiovascular disease risk history or long‐term cardiac medication compared to individuals with no adverse event (Table 2).

**TABLE 1:** Characteristics of patients according to the presence and absence of myocardial ischaemia.

Characteristic	NI (*n* = 220)	IS (*n* = 184)	*P*
Demographics			
Age (years)	70.2 ± 9.9	69.7 ± 10.5	0.67
Men (*n* (%))	121 (55)	122 (66.3)	0.02
Height (cm)	166.8 ± 11.2	167.5 ± 9.5	0.52
Weight (kg)	81.2 ± 19.9	81.3 ± 18.6	0.98
BSA (kg m^−2^)	1.93 ± 0.27	1.93 ± 0.25	0.82
History (*n* (%))			
Hypertension	171 (77.7)	143 (77.7)	0.99
Diabetes mellitus	63 (28.6)	66 (35.9)	0.12
Hypercholesterolaemia	156 (70.9)	155 (84.2)	0.002
Family history of CVD	76 (34.5)	77 (41.8)	0.13
Prior myocardial infarction	51 (23.2)	75 (40.8)	<0.001
PCI	68 (30.9)	83 (45.1)	0.003
CABGS	22 (10)	28 (15.2)	0.11
Smoking history			0.56
Never smoker	110 (50)	87 (47.3)	
Ex‐smoker	88 (40)	79 (42.9)	
Current smoker	20 (9.1)	18 (9.8)	
Long term cardiac medication (*n* (%))			
ACEI	69 (31.4)	82 (44.6)	0.006
Angiotensin II receptor antagonist	40 (18.2)	40 (21.7)	0.37
Aspirin	106 (48.2)	108 (58.7)	0.05
Beta blockers	105 (47.7)	101 (54.9)	0.15
Calcium antagonists	71 (32.3)	53 (28.8)	0.45
Diuretic	28 (12.7)	32 (17.4)	0.19
Nitrates	47 (21.4)	57 (31.0)	0.03
Statin	151 (68.6)	147 (79.9)	0.01
Warfarin	6 (2.7)	6 (3.3)	0.75
Baseline echocardiography data			
IVSd (cm)	1.08 ± 0.21	1.06 ± 0.20	0.19
LVIDd (cm)	4.59 ± 0.62	4.61 ± 0.60	0.67
PWTd (cm)	0.95 ± 0.19	0.94 ± 0.18	0.68
LVIDs (cm)	2.91 ± 0.58	3.02 ± 0.64	0.08
LA size (cm)	3.65 ± 0.69	3.63 ± 0.62	0.72
RWT	0.45 ± 0.09	0.44 ± 0.09	0.20
LV mass (g)	165.5 ± 56.3	164.4 ± 57.9	0.84
LV mass index (g m^−2^)	85.2 ± 24.7	84.2 ± 24.9	0.67
A4C LA volume (ml)	52.1 ± 26.0	49.9 ± 22.0	0.37
A2C LA volume (ml)	53.5 ± 26.8	52.1 ± 22.4	0.56
Mitral *E*	0.64 ± 0.21	0.64 ± 0.22	0.61
Mitral *A*	0.84 ± 0.28	0.80 ± 0.23	0.12
Mitral *E*/*A*	0.83 ± 0.43	0.90 ± 0.61	0.15
Mitral *E* deceleration (ms)	218.9 ± 70.5	211.5 ± 63.8	0.28
Lateral *E*′	0.09 ± 0.03	0.09 ± 0.03	0.53
Lateral *S*′	0.09 ± 0.05	0.08 ± 0.02	0.45
Septal *E*′	0.07 ± 0.02	0.07 ± 0.02	0.07
Septal *S*′	0.07 ± 0.02	0.07 ± 0.02	0.86
*E*/*E*′ lateral	7.5 ± 3.0	8.0 ± 4.0	0.16
*E*/*E*′ septal	9.6 ± 5.5	10.3 ± 5.0	0.17
*E*/*E*′ average	8.5 ± 3.9	9.2 ± 4.2	0.14
TAPSE	2.05 ± 0.47	2.01 ± 0.43	0.32
A4C LVEDV (ml)	100.8 ± 34.8	100.6 ± 34.8	0.93
A4C LVESV (ml)	40.0 ± 21.9	38.7 ± 19.9	0.56
A2C LVEDV (ml)	95.8 ± 33.6	98.8 ± 35.4	0.39
A2C LVESV (ml)	38.2 ± 19.7	36.3 ± 18.1	0.32
Biplane LVEF (%)	61.8 ± 9.4	62.3 ± 9.5	0.24
A4C GLS (%)	−16.2 ± 3.3	−15.5 ± 3.1	0.04
A3C GLS (%)	−15.8 ± 3.4	−15.2 ± 3.4	0.13
A2C GLS (%)	−16.5 ± 3.1	−15.8 ± 3.2	0.03
Average GLS (%)	−16.2 ± 2.7	−15.5 ± 2.9	0.02
DSE test			
Baseline heart rate (b min^−1^)	71.9 ± 13.6	71.7 ± 13.1	0.86
Peak heart rate (b min^−1^)	133.4 ± 11.6	135.1 ± 13.8	0.16
Baseline sBP (mmHg)	137.1 ± 19.7	134.5 ± 17.9	0.18
Peak sBP (mmHg)	149.4 ± 24.5	148.9 ± 23.0	0.84
Baseline dBP (mmHg)	71.8 ± 12.4	70.8 ± 10.9	0.38
Peak dBP (mmHg)	66.5 ± 12.5	65.3 ± 11.2	0.32
Resting WMSI	1.05 ± 0.17	1.08 ± 0.18	0.09
Peak WMSI	1.05 ± 0.17	1.24 ± 0.21	<0.001
Fixed wall motion abnormality	26 (11.8)	44 (23.9)	0.001
Chest pain	13 (5.9)	28 (15.2)	0.002
12‐month follow‐up			
Adverse event (*n* (%))	32 (14.5)	23 (12.5)	0.55

Abbreviations: ACEI, angiotensin convertor enzyme inhibitor; AP2, apical 2‐chamber view; AP4, apical 4‐chamber view; BSA, body surface area; CABGS, coronary artery bypass graft surgery; CVD, cardiovascular disease; dBP, diastolic blood pressure; DSE, dobutamine stress echocardiography; GLS, global longitudinal strain; IVSd, interventricular septal diameter diastole; LA, left atrial; LV, left ventricular; LVEDV, left ventricular end diastolic volume; LVEF, left ventricular ejection fraction; LVESV, left ventricular end systolic volume; LVIDd, left ventricular internal diameter diastole; LVIDs, left ventricular internal diameter systole; NI, non‐ischaemic; PCI, percutaneous coronary intervention; PWTd, posterior wall thickness diastole; sBP, systolic blood pressure; TAPSE, tricuspid annular plane systolic excursion; WMSI, wall motion score index.

**TABLE 2:** Characteristics of patients according to adverse outcomes at 12 months.

Characteristics	No adverse event (*n* = 349)	Adverse event (*n* = 55)	*P*
Demographics			
Age (years)	70 ± 10.1	70 ± 10.5	0.97
Men (*n* (%))	207 (59.3)	36 (65.5)	0.39
Height (cm)	167.2 ± 10.3	166.4 ± 11.1	0.59
Weight (kg)	81.6 ± 19.8	79.1 ± 15.3	0.36
BSA (kg m^−2^)	1.94 ± 0.26	1.91 ± 0.22	0.42
History (*n* (%))			
Hypertension	267 (76.5)	47 (85.5)	0.14
Diabetes mellitus	112 (32.1)	17 (30.9)	0.86
Hypercholesterolaemia	266 (76.2)	45 (81.8)	0.36
Family history of CVD	132 (37.8)	21 (38.2)	0.96
Prior myocardial infarction	106 (30.4)	20 (36.4)	0.37
PCI	134 (38.4)	17 (30.9)	0.29
CABGS	42 (12.0)	8 (14.5)	0.60
Smoking history			0.21
Never smoker	165 (47.3)	34 (61.8)	
Ex‐smoker	150 (43.0)	17 (30.9)	
Current smoker	34 (9.7)	4 (7.3)	
Long‐term cardiac medication (*n* (%))			
ACEI	130 (37.2)	21 (38.2)	0.89
Angiotensin II receptor antagonist	74 (21.2)	6 (10.9)	0.08
Aspirin	183 (52.4)	31 (56.4)	0.81
Beta blockers	173 (49.6)	33 (60.0)	0.15
Calcium antagonists	108 (30.9)	16 (29.1)	0.78
Diuretic	50 (14.3)	10 (18.2)	0.46
Nitrates	84 (24.1)	20 (36.4)	0.05
Statin	255 (73.1)	43 (78.2)	0.42
Warfarin	9 (2.6)	3 (5.5)	0.24
Baseline echocardiography data			
IVSd (cm)	1.07 ± 0.21	1.10 ± 0.22	0.32
LVIDd (cm)	4.61 ± 0.60	4.53 ± 0.70	0.39
PWTd (cm)	0.94 ± 0.19	0.98 ± 0.20	0.09
LVIDs (cm)	2.95 ± 0.60	2.98 ± 0.70	0.81
LA size (cm)	3.64 ± 0.67	3.67 ± 0.60	0.73
RWT	0.44 ± 0.09	0.47 ± 0.12	0.02
LV mass (g)	164.5 ± 56.9	168.4 ± 58.0	0.64
LV mass index (g m^−2^)	84.3 ± 24.6	87.7 ± 25.7	0.34
A4C LA volume (mL)	51.0 ± 24.2	51.8 ± 24.4	0.83
A2C LA volume (mL)	52.6 ± 24.8	54.8 ± 25.7	0.98
Mitral *E*	0.66 ± 0.22	0.60 ± 0.19	0.04
Mitral *A*	0.81 ± 0.26	0.85 ± 0.27	0.36
Mitral *E*/*A*	0.87 ± 0.51	0.82 ± 0.58	0.53
Mitral *E* deceleration (ms)	216.0 ± 67.2	212.5 ± 70.6	0.73
Lateral *E*′	0.09 ± 0.03	0.08 ± 0.03	0.09
Lateral *S*′	0.08 ± 0.04	0.08 ± 0.02	0.61
Septal *E*′	0.07 ± 0.02	0.07 ± 0.03	0.34
Septal *S*′	0.07 ± 0.02	0.07 ± 0.02	0.14
*E*/*E*′ lateral	7.7 ± 3.4	8.0 ± 4.3	0.50
*E*/*E*′ septal	9.8 ± 4.5	10.5 ± 8.9	0.42
*E*/*E*′ average	8.7 ± 3.6	9.3 ± 6.2	0.41
TAPSE	2.04 ± 0.45	1.96 ± 0.48	0.22
A4C LVEDV (mL)	101.6 ± 35.0	95.4 ± 32.9	0.22
A4C LVESV (mL)	39.9 ± 20.8	36.3 ± 22.1	0.24
A2C LVEDV (mL)	97.5 ± 34.7	95.3 ± 33.0	0.66
A2C LVESV (mL)	37.5 ± 18.9	36.2 ± 20.1	0.62
Biplane LVEF (%)	61.5 ± 9.2	63.1 ± 10.3	0.24
A4C GLS (%)	−16.1 ± 3.1	−14.3 ± 3.8	<0.001
A3C GLS (%)	−15.8 ± 3.4	−14.2 ± 3.2	0.002
A2C GLS (%)	−16.3 ± 3.2	−15.1 ± 3.2	0.01
Average GLS (%)	−16.1 ± 2.8	−14.4 ± 2.7	<0.001
DSE test			
Baseline heart rate (b min^−1^)	72.0 ± 13.7	70.8 ± 10.3	0.54
Peak heart rate (b min^−1^)	134.5 ± 12.6	131.8 ± 12.7	0.13
Baseline sBP (mmHg)	135.7 ± 19.3	137.4 ± 16.5	0.54
Peak sBP (mmHg)	148.9 ± 24.3	151.0 ± 20.2	0.54
Baseline dBP (mmHg)	71.0 ± 11.3	73.2 ± 14.0	0.21
Peak dBP (mmHg)	65.5 ± 10.9	68.9 ± 17.1	0.04
Resting WMSI	1.07 ± 0.17	1.07 ± 0.20	0.72
Peak WMSI	1.14 ± 0.21	1.14 ± 0.21	0.81
Fixed wall motion abnormality	60 (17.2)	10 (18.2)	0.86
New wall motion abnormality	161 (46.1)	23 (41.8)	0.55
Chest pain	34 (9.7)	7 (12.7)	0.50
Number of IS LV segments (*n* (%))			0.81
0 LV segments	188 (53.9)	32 (58.2)	
1–2 LV segments	79 (22.6)	12 (21.8)	
≥3 LV segments	82 (23.5)	11 (20.0)	

Abbreviations: ACEI, angiotensin convertor enzyme inhibitor; AP2, apical 2‐chamber view; AP4, apical 4‐chamber view; BSA, body surface area; CABGS, coronary artery bypass graft surgery; CVD, cardiovascular disease; dBP, diastolic blood pressure; DSE, dobutamine stress echocardiography; GLS, global longitudinal strain; IS, ischaemic; IVSd, interventricular septal diameter diastole; LA, left atrial; LV, left ventricular; LVEDV, left ventricular end diastolic volume; LVEF, left ventricular ejection fraction; LVESV, left ventricular end systolic volume; LVIDd, left ventricular internal diameter diastole; LVIDs, left ventricular internal diameter systole; PCI, percutaneous coronary intervention; PWTd, posterior wall thickness diastole; sBP, systolic blood pressure; TAPSE, tricuspid annular plane systolic excursion; WMSI, wall motion score index.

### 3.2 Imaging

There were no significant differences in conventional echocardiographic measures between IS and NI responders (Table 1). However, there were significant differences in longitudinal strain in the apical two‐chamber (*P* = 0.03), four‐chamber (*P* = 0.04) and average GLS (*P* = 0.02) between IS and NI responders. Longitudinal strain was successfully calculated in 86.1% of cases in the two‐chamber view, 90.1% of cases in the four‐chamber view and 86.6% of cases in the three‐chamber view. GLS was successfully calculated in 84% of cases. From the DSE data, unsurprisingly, IS responders had a significantly greater peak WMSI compared to NI responders (*P *< 0.001) and a greater proportion of IS responders had fixed WMAs (*P* = 0.001) and developed chest pain (*P* = 0.002) symptoms during DSE. A greater relative wall thickness (*P* = 0.02) and attenuated mitral E‐wave (*P *= 0.04) were the only conventional echocardiographic measures significantly associated with an adverse event. However, lower longitudinal strain in the apical two‐ (*P* = 0.01), three‐ (*P* = 0.002) and four‐chamber (*P *< 0.001) views, as well as GLS (*P *< 0.001) were significantly associated with an adverse event at 1 year. Peak diastolic blood pressure (*P* = 0.04) was the only DSE parameter associated with an adverse event (Table 2).

### 3.3 Biomarker

**FIGURE 3:**
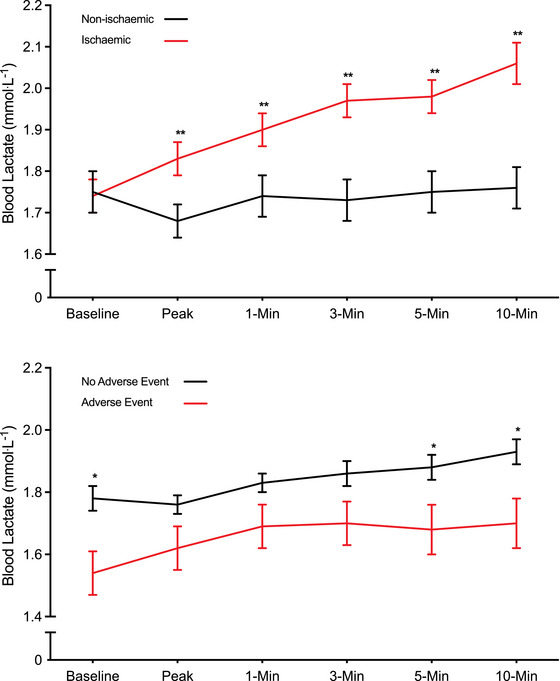
Blood lactate responses to dobutamine stress in the presence and absence of myocardial ischaemia (a), and those with and without an adverse event during follow‐up (b). *P < 0.05 and **P < 0.001 between groups.

**FIGURE 4:**
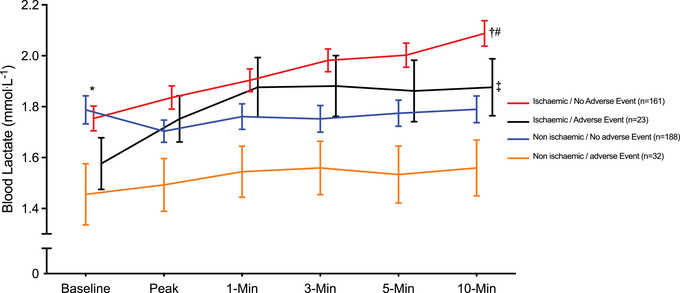
Blood lactate responses to dobutamine stress in the presence and absence of myocardial ischaemia, with and without an adverse event during follow‐up. *P < 0.005 at baseline between those who did and did not have an adverse event. †*P *< 0.001 at 10 min recovery between NI responders with no adverse events and IS responders with no adverse events. #*P *< 0.001 at 10 min recovery between NI responders with adverse events and IS responders with no adverse events. ‡*P* = 0.03 at 10 min recovery and NI responders with adverse events and IS responders with adverse events. IS, ischaemic; NI, non‐ischaemic.

There was no significant difference in [La^−^]_b_ concentrations between IS (1.75 ± 0.76 mmol L^−1^) and NI (1.73 ± 0.60 mmol L^−1^) responders at baseline (*P* = 0.592). However, [La^−^]_b_ concentrations were significantly greater at peak stress (1.83 ± 0.57 vs. 1.68 ± 0.60 mmol L^−1^), 1 (1.90 ± 0.56 vs. 1.73 ± 0.71 mmol L^−1^), 3 (1.97 ± 0.56 vs. 1.73 ± 0.71 mmol L^−1^), 5 (1.98 ± 0.60 vs. 1.74 ± 0.70 mmol L^−1^) and 10 min (2.01 ± 0.63 vs. 1.76 ± 0.71 mmol L^−1^) into recovery in IS compared with NI responders (all *P *< 0.001) (Figure 3a). Attenuated [La^−^]_b_ concentrations at baseline (1.54 ± 0.55 mmol L^−1^ vs. 1.78 ± 0.70 mmol L^−1^, *P* = 0.02), 5 (1.68 ± 0.55 mmol L^−1^ vs. 1.88 ± 0.68 mmol L^−1^, *P* = 0.04) and 10 min (1.70 ± 0.56 mmol L^−1^ vs. 1.93 ± 0.71 mmol L^−1^, *P* = 0.02) into recovery were significantly associated with an adverse cardiac event during 1 year of follow‐up (Figure 3b). A higher IS burden was associated with increased [La^−^]_b_ concentrations at each measurement stage; however, this difference was not statistically significant. In the non‐IS group, there was a significant difference at baseline between those who did and did not have an adverse event (*P* = 0.019) and a trend at peak (*P* = 0.08) and 10 min recovery (*P* = 0.07) (Figure 4). At the 10 min recovery time point, there was a significant difference between conditions (*P *< 0.001). In addition, there was a significant difference between NI responders with no adverse events and IS responders with no adverse events (*P *< 0.001), NI responders with adverse events and IS responders with no adverse events (*P *< 0.001) and NI responders with adverse events and IS responders with adverse events (*P* = 0.03) (Figure 4).

### 3.4 Coronary angiography

During the 1 year follow‐up period, 74 (18.3%) patients underwent coronary angiography (18 with fractional flow reserve) following their DSE. Of these, 46 (62.2%) demonstrated significant CAD (33 single vessel, 7 double vessel and 6 triple vessel disease), requiring coronary intervention (42 percutaneous coronary intervention and 4 coronary artery bypass graft surgery). Patients who underwent angiography had a significantly greater peak wall motion score index (PWSI) (1.27 ± 0.2 vs. 1.11 ± 0.2, *P *< 0.001) on DSE and of the 46 patients requiring coronary intervention, 45 (97.8%) were IS responders.

### 3.5 Clinical outcomes

During the 1 year follow‐up period, 55 (13.6%) patients experienced an adverse event. Table 3 provides a breakdown of the adverse events during the follow‐up period. In multivariate Cox regression, GLS (HR 1.21; 95% CI: 1.11–1.33, *P *< 0.001) and [La^−^]_b_ concentrations at 10 min into recovery (HR 0.54; 95% CI: 0.33 to 0.85, *P* = 0.01) were significant independent predictors of an adverse event.

**TABLE 3:** Summarises TEAEs by SOC, PT and where applicable, the LLT using MedDRA terminology.

SOC/PT/LLT (MedDRA)	Ischaemic (*n* = 184)	Non‐ischaemic (*n* = 220)	Total (*n* = 404)
All SOCs	23 (12.5%) [23]	32 (14.5%) [32]	55 (13.6%) [55]
Cardiac disorders	20 (10.9%) [20]	29 (13.2%) [29]	49 (12.1%) [49]
PT: angina pectoris/chest pain	16 (8.7%) [16]	26 (11.8%) [26]	42 (10.4%) [42]
PT: arrhythmia	2 (1.1%) [2]	0 (0.0%) [0]	2 (0.5%) [2]
PT: cardiac failure	2 (1.1%) [2]	3 (1.4%) [3]	5 (1.2%) [5]
Nervous system disorders	3 (1.6%) [3]	2 (0.9%) [2]	5 (1.2%) [5]
PT: transient IS attack	3 (1.6%) [3]	2 (0.9%) [2]	5 (1.2%) [5]
Vascular disorders			
PT: aortic aneurysm	0 (0.0%) [0]	1 (0.5%) [1]	1 (0.2%) [1]
LLT: aortic aneurysm, abdominal	0 (0.0%) [0]	1 (0.5%) [1]	1 (0.2%) [1]

*Note*: Data are stratified by ischaemic and non‐ischaemic responders. Abbreviations: %, percentage of group affected; [e], number of events (assuming one event per subject); Events coded using MedDRA version 28.0; IS, ischaemic; LLT, lowest level term; *n*, number of subjects affected; PT, preferred term; SOC, system organ class; TEAEs, treatment‐emergent adverse events.

## 4 DISCUSSION

Cardiac stress testing provides important diagnostic and prognostic information in patients with suspected IS heart disease. This is the largest study to assess transient alterations in a circulating metabolic biomarker in response to dobutamine stress. Our results demonstrate a significantly elevated [La^−^]_b_ response in patients with myocardial ischaemia, which extends into the recovery period. The difference in [La^−^]_b_ concentrations reported are greater than the analytical reliability of the blood Lactate Pro 2 device, which supports these being true physiological alterations.

Previous research has demonstrated that acidosis occurs in the IS heart of patients during atrial pacing. Of particular interest was the reported pronounced change during the recovery (washout) period following pacing (Cobbe & Poole‐Wilson, 1982). This novel study using dobutamine stress demonstrated a similar response, where elevations in [La^−^]_b_ concentrations peak at 10 min into recovery with a plateau response in the NI heart. Myocardial ischaemia with dobutamine is only transient; however, the significant increase in [La^−^]_b_ concentrations in IS responders at peak stress with no change in NI responders indicates there must be an efflux of anaerobic metabolites from the IS heart. The continued elevation in [La^−^]_b_ concentrations during the recovery period may be due to the re‐perfused IS territory washing metabolic waste into the circulation (Cobbe & Poole‐Wilson, 1982). The evidence of no change in NI responders indicates the absence of any metabolic alterations. In fact, there is a small decrease in [La^−^]_b_ concentrations at peak stress in NI responders, which suggests the normal heart may use circulating [La^−^]_b_ as a metabolic fuel during stress as a result of increased workload (Brooks, 2021). Indeed, previous research demonstrates that the ratios of high‐energy phosphates remain unchanged in the NI heart during increased cardiac work in humans (Weiss et al., 1990). These results are supported by animal studies over a range of cardiac (from 5000 to 25,000 mmHg b min^−1^) workloads (Balaban et al., 1986). Thus, in the absence of a compromised coronary blood supply, the myocardium is able to regulate and maintain high‐energy phosphate levels over a range of workloads. However, in the IS heart, there was a decrease in the phosphocreatine to ATP ratio, reflecting a transient imbalance between oxygen supply and demand in the myocardium with a compromised blood flow (Weiss et al., 1990). The resolution of these responses in a sub‐group of patients who underwent revascularisation, along with the absence of change in the NI heart, suggests these findings are true metabolic alterations specific for the IS myocardium. Furthermore, despite severe chest pain and reduced coronary flow reserve after pacing, patients with angina and a normal coronary angiogram do not demonstrate metabolic evidence of myocardial ischaemia (Rosano et al., 1996).

Our findings may be of clinical importance since [La^−^]_b_ concentrations >2 mmol L^−1^ was associated with a 1.94‐ to 10.89‐fold increased risk of mortality in patients admitted to intensive care (Khosravani et al., 2009). Although our cohort of participants are outpatients and long‐term outcome is currently unknown, it may be that IS responders with [La^−^]_b_ concentrations >2 mmol L^−1^ in recovery require intensified medical management and/or coronary angiography for the investigation of significant CAD, independent to the burden of ischaemia on functional stress testing.

Previous work has demonstrated different biomarker responses for high sensitivity cardiac troponin T (hs‐cTnT) and B‐type natriuretic peptide signal peptide (BNPsp) in response to dobutamine infusion in a small population of healthy (*n* = 10), IS (*n* = 13) and NI (*n* = 3) responders. These biomarkers could differentiate IS and NI responders (Siriwardena et al., 2012). In addition, Ikonomidis et al. (2005) demonstrated that reversible myocardial ischaemia provoked by DSE (*n* = 103) caused a significant increase in interleukin‐6 and tissue factor plasma levels. However, biomarker sampling was performed up to 240 and 30 min post‐infusion, respectively, and samples required laboratory analysis. Analysis of [La^−^]_b_ concentrations is inexpensive and can be performed in real time in the stress echo laboratory without delaying patient time. Our results suggest [La^−^]_b_ responses reflect the short‐lived ischaemia in patients undergoing DSE for the evaluation of cardiac chest pain. As such, routine sampling may improve the overall utility of DSE. However, further multi‐centre research with appropriate follow‐up is required to test this hypothesis.

The significantly lower [La^−^]_b_ concentrations at baseline and in response to stress in participants who experienced an adverse event during the follow‐up period may be due to differences in myocardial work, which influences cardiac metabolism. Indeed, changes in cardiac work determine the rates and relative use of energy substrate, with lower work associated with lipid oxidation and greater work associated with carbohydrate energy sources, which may influence contractility (Stanley et al., 2005). When cardiac work is elevated, the myocardium relies heavily on lactate as a fuel (Brooks, 2021), and alterations in cardiac metabolism as a result of heart disease can ultimately impair the myocyte contractile proteins in a sequela of events, which leads to a decreased responsiveness of the contractile proteins to activator calcium (Lopaschuk et al., 2010). The significantly lower GLS, which is a sensitive marker of myocardial mechanical contraction, and significantly greater adverse LV remodelling in those that experienced an adverse event supports this concept. Animal model research has demonstrated that systemic lactate deprivation is detrimental to myocardial energetics and cardiovascular performance and is associated with adverse outcomes (Barbee et al., 2000; Levy et al., 2007). Importantly, GLS and [La^−^]_b_ concentration at 10 min of recovery were independent predictors of an adverse event at 1 year. In addition, when analysing the data based on IS and NI responses with and without adverse events (Figure 4), an important observation was that the NI and no adverse event group was the only group to see a reduction in [La^−^]_b_ from baseline to peak dose dobutamine. In addition, of the IS responders who experienced an adverse event, [La^−^]_b_ concentrations plateaued after 1 min of recovery, whereas, in IS responders without an adverse event, [La^−^]_b_ continued to increase up to 10 min into recovery. It would be of interest to assess the peak DSE images to understand if GLS in IS responders is depressed compared to NI responders (O'Driscoll et al., 2022), since accumulation of [La^−^]_b_ is associated with a fall in intracellular pH, which impairs myofilament and sarcoplasmic reticulum pump function (Fabiato & Fabiato, 1978) and is associated with Ca^2+^ overload (Murphy et al., 1991).

DSE involves therapeutically inducing a supply–demand mismatch, and it is noteworthy that most previous research has reported that elevation in [La^−^]_b_ is associated with increased mortality in critically ill patients. This is evidenced in emergency settings (Gatien et al., 2005) including cardiogenic shock (Chiolero et al., 2000), myocardial infarction (Lazzeri et al., 2012; Vermeulen et al., 2010) and renal disease (Koreny et al., 2002). However, this study included ambulatory outpatients referred for a DSE for investigation of chest pain. As such, the lower [La^−^]_b_ concentrations and attenuated response to dobutamine stress in patients who experienced an adverse event requires further research. Of interest, mean [La^−^]_b_ concentrations at peak and in recovery in IS responders was similar to the highest risk group cut‐offs in a study reporting admission [La^−^]_b_ concentrations in patients with myocardial infarction (Vermeulen et al., 2010).

### 4.1 Limitations

This was a single centre study of patients referred for a clinically indicated SE. Myocardial metabolism is complex and difficult to study in humans. As such, verification of these findings is required. In this study we used DSE given that it is a reproducible, standardised, non‐invasive clinical technique used to analyse transient myocardial ischaemia. Importantly, dobutamine infusion enables isolated stress of the cardiac muscle in a stationary position, which reduces influences from other organ systems on [La^−^]_b_ concentrations. The Lactate Pro 2 device was not available for all patient lists and this may explain the higher proportion of IS responders in this study. We selected to record [La^−^]_b_ concentrations up to 10 min into recovery, which in this study shows the highest [La^−^]_b_ concentrations. Therefore, it is unknown what happens beyond 10 min of recovery time. Additional data acquisition may have provided a stronger independent prediction of adverse events (optimal threshold) and/or the time it takes for [La^−^]_b_ concentrations to return to basal levels. Although adjusted in our analysis, differences in medication status may impact lactate responses. Finally, hydration status was not assessed, which may influence [La^−^]_b_ concentrations relative to blood volume. Dehydration reduces blood volume, impairing oxygen and nutrient delivery to muscles, which can lead to earlier lactate accumulation. Additionally, lower plasma volume due to dehydration results in less dilution of lactate, potentially increasing its concentration in the blood. Dehydration can also reduce lactate clearance by affecting kidney function and acid–base balance. Thus, without assessing hydration, lactate concentrations may not accurately reflect the true metabolic response.

### 4.2 Conclusion

Transient myocardial ischaemia is associated with a significant elevation in [La^−^]_b_ concentrations, which extends into the recovery period. A blunted metabolic response and attenuated longitudinal myocardial mechanics are independently associated with short‐term adverse events. The ability of routine sampling of [La^−^]_b_ to improve the overall utility of DSE requires further multi‐centre research with appropriate follow‐up time.

## AUTHOR CONTRIBUTIONS

Jamie M. O'Driscoll, Anna Marciniak, and Rajan Sharma conceptualized the study. Jamie M.O'Driscoll, Elliot Smith, Matchel Bibat, Jamie J. Edwards, Claire Compton, Konstantina Kipourou, and Eliane Cunliffe was involved in patient recruitment and data acquisition. Damian Coleman conducted statistical analyses. Jamie M.O'Driscoll and Rajan Sharma wrote the first draft of the manuscript. Jamie M.O'Driscoll, Elliot Smith, Matchel Bibat, Jamie J. Edwards, Claire Compton, Konstantina Kipourou, Damian Coleman, Eliane Cunliffe, Anna Marciniak, and Rajan Sharma, contributed to the interpretation of data and all authors critically revised the manuscript. All authors have read and approved the final version of this manuscript and agree to be accountable for all aspects of the work in ensuring that questions related to the accuracy or integrity of any part of the work are appropriately investigated and resolved. All persons designated as authors qualify for authorship, and all those who qualify for authorship are listed.

## CONFLICT OF INTEREST

None declared.

### FUNDING INFORMATION

None.

## Data Availability

The data that support the findings of this study are available from the corresponding author upon reasonable request.
